# Preparation of Crosslinked Gelatin Microparticles and Study on Their Loading Capacity for Folic Acid

**DOI:** 10.3390/polym17212815

**Published:** 2025-10-22

**Authors:** Jia-Yi Qi, Xiao-Feng Hu, Dan Qiu, Ya-Juan Wang, Zhang-Fa Tong

**Affiliations:** 1 University Engineering Research Center of Green Chemical New Materials, School of Chemistry and Chemical Engineering, Guangxi University, Nanning 530004, China; qjyen2023@163.com (J.-Y.Q.); zhftong@sina.com (Z.-F.T.); 2 School of Biological and Chemical Engineering, NingboTech University, Ningbo 315100, China; 3 Ningbo Jildan Health Science and Technology Co., Ltd., Ningbo 315200, China; huxiaofeng@jildan.com; 4 School of Materials and Chemical Engineering, Ningbo University of Technology, Ningbo 315211, China; 5 Zhejiang Institute of Tianjin University, Ningbo 315201, China

**Keywords:** crosslinked gelatin microparticles (cGMPs), tea polyphenols, thermal stability, folic acid, in vitro digestion

## Abstract

Gelatin microparticles (GMPs) can load functional active substances, but they tend to redissolve in high-temperature aqueous solutions during food processing. In this study, a new loading system adapted to food processing and digestive environments was constructed through the crosslinking of tea polyphenols (TP) on GMPs. The effects of pH, temperature, and crosslinking time on the methylene blue (MB) retention rate in crosslinked gelatin microparticles (cGMPs) were investigated, resulting in optimized crosslinking conditions. Compared with GMPs, the surface of cGMPs was denser and smoother. ATR-FTIR results showed that the N–H groups were involved in the formation of hydrogen bonds during the crosslinking process. The crosslinking effect of TP significantly disrupted the triple-helical structure of gelatin. The melting temperature ($T_{m}$) of cGMPs is 147.79 °C, which is significantly higher than that of GMPs (87.11 °C), indicating a marked improvement in thermal stability. In high-temperature aqueous solutions, Folic acid-loaded cGMPs (FA-cGMPs) maintained morphological integrity for 2 h (at 40 °C) and 0.5 h (at 60 °C). In vitro digestion simulations revealed excellent sustained-release characteristics of FA-cGMPs, with a release rate of only 4.91% in simulated gastric fluid and 88.13% in simulated intestinal fluid. This study provides an ideal carrier with food processing stability and intestinal-targeted release capabilities for functional active substances.

## 1. Introduction

Gelatin, a natural hydrophilic polymer derived from the partial hydrolysis of collagen, exhibits remarkable advantages as a natural biomaterial. It demonstrates excellent biocompatibility [1] due to its structural similarity to biological tissues, ensuring favourable host acceptance. Moreover, gelatin possesses inherent biodegradability [2], with non-toxic and easily absorbable degradation products. Its outstanding film-forming capability and adhesiveness as well as emulsifying, foaming, stabilising and clarifying properties [3] significantly broaden its application potential. These superior characteristics, combined with low production costs, make gelatin widely used in the food, pharmaceutical and chemical industries.

Gelatin microparticles (GMPs) have gained extensive attention in the food and pharmaceutical industries owing to their facile preparation, low cost, high drug-loading capacity, excellent dispersibility, good histocompatibility and biodegradability [4,5]. GMPs can be formed directly through physical methods including freeze-drying and spray-drying [6,7]. However, such physically crosslinked GMPs tend to redissolve in high-temperature aqueous solutions, limiting their applicability under certain processing conditions [8]. Chemical crosslinking can markedly enhance microparticle formation efficiency, mechanical properties, and water resistance [9]. For instance, De Clercq et al. [10] developed genipin-crosslinked GMPs for preventing postoperative peritoneal adhesions, where adjusting genipin concentration and crosslinking duration controlled microparticle residence time while improving thermal and structural stability. Similarly, Sabbagh et al. [11] fabricated microneedles using glutaraldehyde-crosslinked GMPs combined with catechin-loaded poly (vinyl alcohol) (PVA) solution, demonstrating that chemical crosslinking reinforced microparticle structure and enhanced stability. Nevertheless, chemically crosslinked GMPs face limitations in food and pharmaceutical applications due to the potential cytotoxicity of residual crosslinkers [12] and incomplete in vivo degradation of highly crosslinked microparticles [13]. Notably, natural antioxidants like tea polyphenols (TP) exhibit crosslinking effects with gelatin [14], suggesting that TP modification can improve GMPs stability in high-temperature aqueous environments.

Folic acid (FA), a highly bioactive water-soluble compound, has been widely used in food and pharmaceutical applications. However, processing steps involving high-temperature aqueous environments (e.g., in gummy candy and beverage production) lead to significant FA degradation (40–70% loss) [15,16,17], representing a major technical challenge. Recent encapsulation approaches have demonstrated improved stability: Fathima et al. [18] developed chitosan nanoparticles for FA loading, which showed enhanced stability compared with free FA. Fang et al. [19] fabricated glutaraldehyde-crosslinked pea globulin nanoparticles that enabled controlled release and efficient delivery of FA. Salević-Jelić et al. [20] successfully encapsulated FA in composite zein/pumpkin seed protein concentrate/alginate nanoparticles, markedly improving antioxidant activity and storage stability. Although these studies confirm that encapsulation enhances FA stability, research on encapsulation systems capable of withstanding high-temperature aqueous processing remains limited.

Based on previous research [21], this study prepared GMPs via a dropping method, followed by surface modification with TP to obtain crosslinked GMPs (cGMPs) with optimized structural integrity. The resulting cGMPs were evaluated for their FA loading capacity and stability under high-temperature aqueous processing conditions and in vivo digestive environments. This approach aimed to establish an effective loading method for highly active water-soluble functional factors, thereby facilitating broader applications of gelatin and FA in the food and pharmaceutical fields.

## 2. Materials and Methods

### 2.1. Materials

Porcine gelatin (bloom 250) was purchased from Rousselot (Wenzhou, China) Gelatin Co., Ltd. Food-grade medium-chain triglycerides (MCT) were purchased from Shandong Usolf Chemical Technology Co., Ltd. (Linyi, China). Green tea polyphenols (TP, AR) was purchased from Zhejiang Delekang Food Co., Ltd. (Taizhou, China). Porcine pepsin, methanol (HPLC), methylene blue (MB, AR) and bile salts were obtained from Aladdin Reagent Co., Ltd. (Shanghai, China). FA (AR) was purchased from Shanghai Maclean Biochemical Technology Co., Ltd. (Shanghai, China). Anhydrous ethanol (AR) was purchased from Shanghai Titan Technology Co., Ltd. (Shanghai, China). Anhydrous sodium carbonate (Na_2_CO_3_, AR), sodium hydroxide (NaOH, AR), potassium dihydrogen phosphate (KH_2_PO_4_, AR), dipotassium phosphate (K_2_HPO_4,_ AR) and sodium chloride (NaCl, AR) were purchased from Sinopharm Chemical Reagents Co., Ltd. (Shanghai, China). Trypsin was purchased from Beijing Solarbio Science & Technology Co., Ltd. (Beijing, China). All chemical reagents can be used directly in experiments without any purification.

### 2.2. Preparation of Microparticlees

#### 2.2.1. Preparation of GMPs

To obtain a gelatin water solution, approximately 4.95 g of gelatin and 20 mL of ultrapure water were added into a 50-mL beaker and stirred at 60 °C until gelatin completely dissolved. This gelatin solution was drawn with a syringe into cooled MCT (−10 °C), and a needle with an inner diameter of 0.29 mm was used. The coagulation bath was pre-cooled in an ultra-low temperature freezer (−80 °C) and then freeze-dried for 8 h. After freeze-drying, the insoluble substances were aspirated and the residual MCT on the surface of the microparticles was washed off with anhydrous ethanol and then freeze-dried to obtain the final GMPs.

#### 2.2.2. Preparation of cGMPs

The GMPs, prepared as described in Section 2.2.1, were crosslinked by immersing and stirring them in a TP solution (3 g of TP dissolved in 20 mL of ultrapure water, pH adjusted to 4.5). The crosslinking was allowed to proceed for 18 h at 35 °C under constant magnetic stirring (1200 rpm). After the crosslinking was completed, cGMPs were obtained via vacuum filtration, thoroughly washed with ultrapure water, and then freeze-dried to obtain the final sample.

**Scheme 1:**
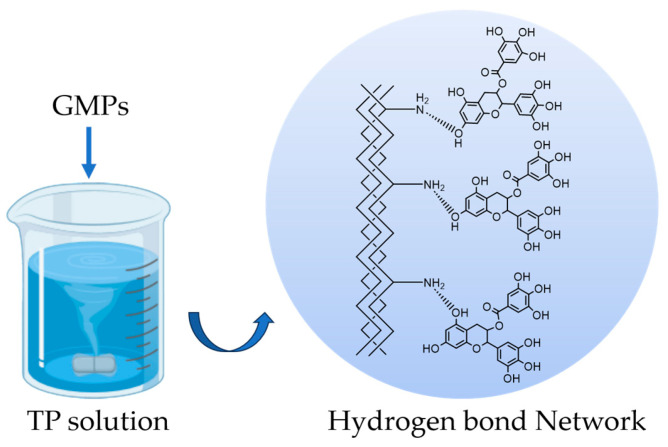
Schematic illustration of the proposed hydrogen-bonding crosslinking mechanism between gelatin and TP. The phenolic hydroxyl groups (-OH) on TP form a hydrogen bond network with -NH_2_ groups on the gelatin chains.

The proposed crosslinking mechanism between gelatin and TP, primarily through hydrogen bonding, is illustrated in Scheme 1.

### 2.3. Preparation of Substrate-Loaded cGMPs

#### 2.3.1. Preparation of MB-Loaded cGMPs (MB-cGMPs)

MB is effective for evaluating the encapsulation ability of microparticles [22,23]. Approximately 4.95 g of gelatin and 20 mL of deionised water were added into a 50-mL beaker and stirred at 60 °C until completely dissolved. Then, the stirring temperature was reduced to 45 °C, and 0.05 g of MB was added and stirred well to prepare an MB–gelatin mixture. This mixture was drawn with a syringe, and an MCT (oil phase) pre-cooled to −10 °C was used as the coagulation bath. A needle with an inner diameter of 0.29 mm was used to manually drop the solution into the coagulation bath at a constant speed to form balls. After balling, the microparticle-containing coagulation bath was transferred to an ultra-low temperature freezer at −80 °C overnight for pre-freezing, followed by freeze-drying for 8 h. After freeze-drying, MB-loaded GMPs (MB-GMPs) were collected via vacuum filtration and washed three times with anhydrous ethanol to thoroughly remove residual MCT on the surface of the microparticles. MB-GMPs were then placed in a fume hood to dry. The dried MB-GMPs were immersed in a TP solution for crosslinking under varying conditions (temperature: 25–45 °C; time: 6–30 h; pH: 4.0–6.0). Among these, the combination of 35 °C, 18 h, and pH 4.5 was identified as optimal, as it yielded the highest MB retention rate, indicating the most effective crosslinking (Figures S1–S3). The process was conducted under magnetic stirring (1200 rpm) and ensured that the microparticles did not stick together. After the crosslinking was completed, MB-GMPs were obtained via vacuum filtration, thoroughly washed with ultrapure water, and then freeze-dried to obtain the final sample MB-cGMPs.

#### 2.3.2. Preparation of FA-Loaded cGMPs (FA-cGMPs)

Given the small amount of FA added to food, this study aimed to improve its stability by directly preparing FA-loaded GMPs (FA-GMPs) based on the maximum MB retention rate condition determined by previous optimisation (Figures S1–S3). First, the FA solution was prepared by dissolving 0.5 g of FA in 15 mL of 0.01 M sodium hydroxide solution and ultrasonically treating until the solution became clear. Then, in a 50-mL beaker, 4.95 g of gelatin and 18.5 mL of ultrapure water were added and stirred at 60 °C until the gelatin was completely dissolved. After lowering the system temperature to 45 °C, 1.5 mL of the above FA solution was added and stirred well to obtain a gelatin–FA mixture. Subsequent procedures are described in Section 2.3.1.

### 2.4. Determination of the MB Retention Rate

A wavelength-increasing scan of the MB solution, performed using a Cary 60 ultraviolet spectrophotometer (Agilent Technologies, Santa Clara, CA, USA), yielded a maximum absorption wavelength of 660 nm for MB.

A standard stock solution of MB was prepared at a concentration of 1000 µg/mL. A series of standard working solutions were then obtained by, respectively, transferring 0.05, 0.1, 0.2, 0.3, 0.4, and 0.5 mL of the stock solution into 50-mL volumetric flasks, followed by dilution to the mark with ultrapure water. The standard curve for MB was established using a series of standard solutions with concentrations of 1, 2, 4, 6, 8, and 10 µg/mL. The standard calibration curve was generated by plotting absorbance against concentration and performing linear regression.

MB standard curve (Figure S4):

(1) Y=0.1023X+0.00176

Here, *Y* represents absorbance and *X* represents MB concentration (µg/mL). The correlation value of the equation was R^2^ = 0.999.

Each of the two MB-cGMPs samples (50 mg each) was weighed in a brown sample bottle. Then, 20 mL of deionised water was added to one sample. Moreover, pepsin was added at 2000 U/g sample, dissolved, and the pH was adjusted to 2.0. Then, the mixture was enzymatised in a 36 °C water bath to complete dissolution. The entire solution was transferred to a 50-mL volumetric flask to make up the volume. Approximately 20 mL of deionised water was added to another sample and stirred and heated at 60 °C for 30 min. After centrifugation, 10 mL of the supernatant was measured in a 50-mL volumetric flask to make up to volume and freeze-dried; subsequently, the undissolved portion was weighed. The absorbances measured under these two different conditions represent the amount of MB released, and are denoted as *I*_1_ (after enzymatic digestion) and *I*_2_ (after heat treatment). Using blank GMPs prepared under the same conditions as reference, the absorbance of the two volumetric solutions was measured at a wavelength of 660 nm and the values were recorded as $I_{1}$ and $I_{2}$. The *MB retention rate* was calculated using Formula (2):

(2) $$MB\;retention\;rate=\frac{I_{1}-I_{2}}{I_{1}}\times 100\%$$

### 2.5. Determination of FA Concentration

A standard stock solution of FA was prepared at a concentration of 10 µg/mL. A series of standard working solutions were then obtained by, respectively, transferring 0, 0.5, 1, 5, and 10 mL of the stock solution into 10-mL volumetric flasks, followed by dilution to the mark with 0.01 M NaOH solution. The standard curve for FA was established using a series of standard solutions with concentrations of 0, 0.5, 1.0, 5.0, and 10.0 µg/mL. The standard calibration curve was generated by plotting the HPLC peak area against concentration and performing linear regression.

FA standard curve (Figure S5):

(3) Y=65.359X−4.043

Here, *Y* represents the peak area (mAW), *X* represents FA concentration (μg/mL), and R^2^ = 0.999. The HPLC system used was the Agilent 1260 series (Agilent Technologies, Santa Clara, CA, USA) with Nouryon Kromasil C18 column. The mobile phase was methanol: 0.02 mol/L K_2_HPO_4_ (pH = 3) = 20:80 (*V*/*V*); flow rate 0.5 mL/min; column temperature 30 °C. The detection wavelength was set to dual-wavelength 278 and 220 nm, with an injection volume of 10 μL.

After preparing 0.01 M NaOH solution, 20 mL of it was taken and mixed with 0.05 g of FA-cGMPs. This mixture was ultrasonicated for 5 min. Then, the pH of the solution was adjusted to 8, and 0.05 g of neutral protease was added to dissolve and obtain the enzyme solution. This solution was stirred and enzymatised at 30 °C for 12 h. After complete enzymatic hydrolysis, the pH was adjusted to 3 to inactivate the enzyme. Then, the pH was adjusted to 8 and the volume was made up to 50 mL with 0.01 M NaOH. After adjusting the pH to 11–12, the solution was shaken well, and the clearant was filtered through a 0.45-μm aqueous phase filter membrane for testing.

### 2.6. Effect of Crosslinking Conditions on MB Retention Rate

This experiment utilized a single-factor design to investigate the effects of pH, temperature, and crosslinking duration on the MB retention rate in GMPs. The concentration of the TP solution was fixed at 3 g per 20 mL of ultrapure water, and the gelatin concentration was maintained at 20%. The specific experimental conditions included pH (4.0, 4.5, 5.0, 5.5, 6.0), temperatures (25 °C, 30 °C, 35 °C, 40 °C, 45 °C) and crosslinking durations (6 h, 12 h, 18 h, 24 h, 30 h). The individual impact of each factor on the MB retention rate was systematically evaluated.

### 2.7. Characterization Methods

#### 2.7.1. Scanning Electron Microscopy (SEM)

GMPs, cGMPs, and their cross-sections were observed using a Phenom Pro scanning electron microscope (Phenom-World, Eindhoven, The Netherlands). Samples were spread as evenly as possible over the conductive band on the stage, and the remaining sample was blown off. GMPs and cGMPs were coated with gold for 20 s using a SBC-12 ion sputtering spreader (Beijing Scientific Instrument Co., Ltd., Beijing, China) and placed in an SEM to observe and photograph morphological changes.

#### 2.7.2. Attenuated Total Reflection Fourier Transform Infrared Spectroscopy (ATR-FTIR)

Infrared structures of GMPs and cGMPs were determined using a Thermo Fisher Scientific Nicolet iS5 Fourier transform infrared spectrometer (Thermo Fisher Scientific, Waltham, MA, USA) with a diamond single reflection ATR accessory. The following parameters were used: spectral range: 4000–600 cm^−1^, resolution: 4 cm^−1^, and number of scans: 32. The background was an empty crystal scan without sample placement (parameters consistent with the sample). The sample was a solid powder placed over the ATR crystal, with moderate pressure applied to get the sample in close contact with the crystal.

#### 2.7.3. X-Ray Diffraction (XRD)

The crystal structures of GMPs and cGMPs were characterised using a D8 Advance X-ray diffractometer (Bruker AXS GmbH, Karlsruhe, Germany) operated under the following conditions: Cu-Kα radiation at 40 kV and 40 mA, with a scanning speed of 5°/min and a 2θ diffraction angle range of 5–70°. The samples were measured directly as intact microparticles without being ground into powder.

#### 2.7.4. Differential Scanning Calorimetry (DSC)

Melting temperatures ($T_{m}$) and melting enthalpies (ΔH) of GMPs and cGMPs were measured using a DSC-Q2000 differential scanning calorimeter (TA Instruments, New Castle, DE, USA). Approximately 5 mg of the sample was weighed and transferred to 60 µL of an aluminium crucible, using an empty aluminium crucible as a reference. The sample tray was heated at a rate of 10 °C/min from 40 °C to 200 °C in a nitrogen atmosphere.

### 2.8. Stability Testing of FA-cGMPs in High-Temperature Aqueous Solutions

FA-cGMPs stability was evaluated in an aqueous solution at high temperatures, with FA-GMPs as the control. Three samples (0.1000 g each) of FA-GMPs and FA-cGMPs were weighed and placed in 20 mL of ultrapure water at 40 °C, 60 °C, and 80 °C. The samples were taken at 0, 0.5, 1, 1.5, and 2 h. In addition, 5 mL of the release medium was removed each time, and an equal volume of blank release medium (ultrapure water at the same temperature) was immediately replenished. FA release in the sample was determined via HPLC.

If the microparticles completely disintegrated at the measurement point, the instantaneous concentration of FA was determined; if there were still remaining microparticles at the measurement point, the cumulative release rate of FA was calculated.

### 2.9. In Vitro Digestion Protocol of FA-cGMPs

In vitro simulation digestion of FA-cGMPs was performed with FA-GMPs as the control. The formulation of the simulated gastrointestinal fluid was modified with reference to Zhang et al.’s [24] method.

Gastric stage: Microparticles (40 mg) were mixed with 10 mL of simulated gastric fluid (SGF, 2 g NaCl, 3.2 g pepsin, 1 L, pH = 2.0) and then digested in a constant temperature incubator (37 °C, 120 rpm) for 2 h. After the gastric stage was completed, 2 mL of the digestion fluid was sampled to measure FA release. After removal, an equal amount of SGF was replenished.

Intestinal stage: The pH of the digestive fluid was adjusted to 7.4 after the gastric stage. Then, an equal volume of the simulated intestinal fluid (SIF, 2 g trypsin, 8.8 g NaCl, 6.8 g KH_2_PO_4_, 5 g bile salts, 1 L, pH 7.4) was added and mixed. The digestion reaction was continued in a constant temperature incubator for 46 h. The sampling time points were strategically selected to capture key phases of the intestinal release. Samples (4 mL) were collected at 9, 22, 34, and 46 h for analysis, with an equal volume of SIF replenished after each sampling. The initial point at 9 h was chosen based on preliminary studies which observed a critical morphological transition where the microparticles began to flatten and aggregate. Subsequent points were set at approximately equal intervals to monitor the release profile throughout the remaining period. All collected samples were immediately placed in an ice bath to terminate trypsin hydrolysis.

Determination of FA accumulative release rate: 2 mL of digestion fluid was collected from the gastric stage and 4 mL from the intestinal stage. Each sample was diluted to 10 mL using 0.01 M NaOH. The pH of the diluted digest was adjusted to 11–12, filtered through a 0.45 μm needle filter, and the FA content was determined via HPLC.

### 2.10. Statistical Analysis

All experiments were performed in triplicate, and the results are presented as mean ± standard deviation.

## 3. Results and Discussion

### 3.1. Optimisation of the cGMPs Preparation Process

Based on the single-factor experimental results (Figures S1–S3), the optimal preparation conditions for cGMPs were determined as follows: crosslinking duration of 18 h, temperature of 35 °C, and pH 4.5. All subsequent cGMS used for comparative characterization were prepared under these optimized conditions.

### 3.2. Characterization Results

#### 3.2.1. SEM

**Figure 1:**
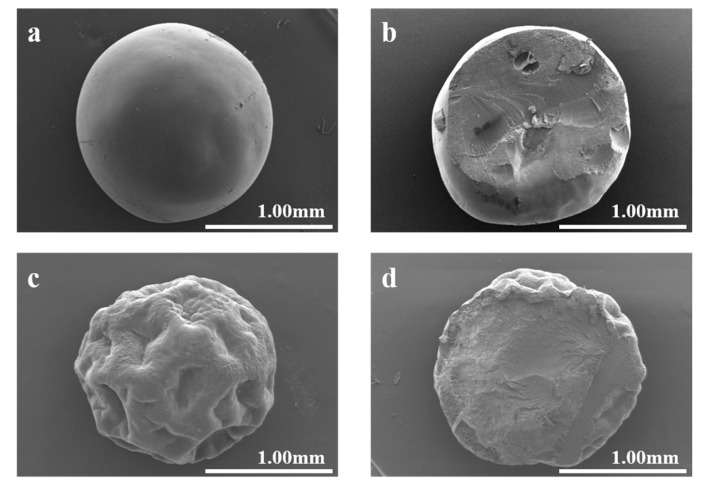
(**a**) cGMP SEM image and (**b**) cross-section; (**c**) GMP SEM image and (**d**) cross-section.

Figure 1 depicts the SEM images of GMPs and cGMPs. Compared with GMPs, the cGMPs’ surface showed denser and smoother characteristics, as obvious depressions were observed for GMPs. This phenomenon occurred because cGMPs can fully absorb water during crosslinking, and the water distribution is uniform. Concurrently, the cross-linked network provides adequate structural integrity, allowing it to maintain a smooth surface shape after subsequent freeze-drying treatment. In contrast, the uncrosslinked microparticles—which could only be briefly soaked (2 min) to avoid dissolution—lacked sufficient water absorption and structural support from a crosslinked network. This deficiency led to their surface collapse and irreversible structural failure during the swelling and freeze-drying processes [25].

The average diameters of the prepared microparticles were statistically analyzed from the SEM images. The results indicated that cGMPs had an average diameter of 1.58 ± 0.087 mm, while GMPs showed an average diameter of 1.61 ± 0.026 mm. The slight difference in size between the two is statistically negligible, demonstrating that the cross-linking process under the employed conditions did not significantly alter the macroscopic dimensions of the microparticles. This finding suggests that the cross-linking primarily affects the internal network structure and stability rather than the overall particle size.

#### 3.2.2. ATR-FTIR Spectroscopy

**Figure 2:**
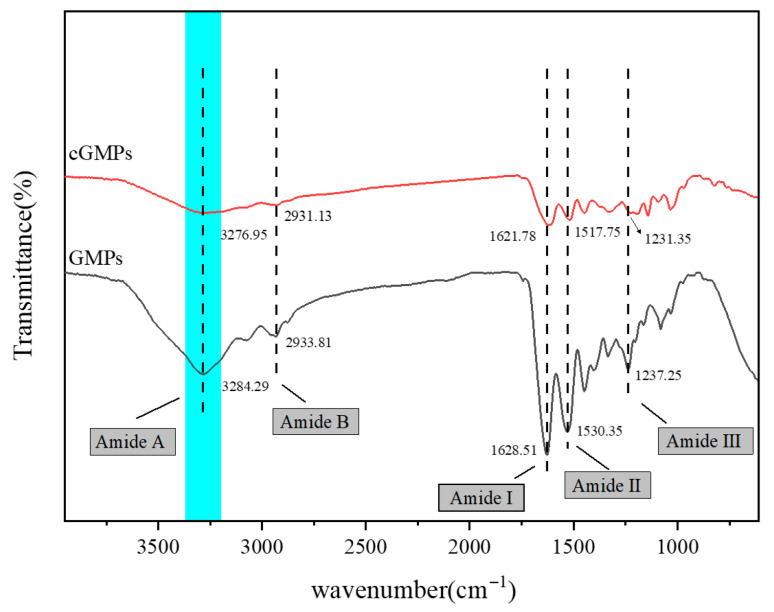
Infrared spectra of GMPs and cGMPs. The highlighted region (Amide A) exhibits a redshift in cGMPs, which confirms the formation of extensive hydrogen bonding between GMPs and TP.

Figure 2 depicts the ATR spectra of GMPs and cGMPs. For GMPs, the characteristic peaks observed at 3284.49, 2933.81, 1621.51, 1530.35, and 1237.25 cm^−1^ were assigned to amide A (O–H or N–H stretching vibration), amide B (–CH_2_- asymmetric stretching vibration), amide I (C=O stretching vibration), amide II (coupling of N–H in-plane bending and C–N stretching vibrations, along with –CH_2_- in-plane bending vibration), and amide III (N–H bending and C–N stretching vibrations), respectively.

Notably, the amide A band of cGMPs, prepared via TP crosslinking, shifted to 3276.95 cm^−1^. This redshift indicates enhanced hydrogen bond formation involving N–H groups during crosslinking [26]. The interaction arises between N–H in gelatin and –OH in TP: when the N–H in –CONH_2_ participates in hydrogen bonding, the amide A band undergoes a redshift [27]. Furthermore, compared with GMPs, the absorption peaks of the amide I and amide II bands in cGMPs appeared at 1621.78 and 1517.75 cm^−1^, respectively, both exhibiting redshifts, which further confirms hydrogen bond formation between TP and GMPs.

The crosslinking effect is therefore attributed to the extensive hydrogen bonding between the phenolic hydroxyl groups of TP and the functional groups on gelatin, as evidenced by the observed redshifts. The aromatic ring structure of TP serves as a crucial rigid scaffold that positions these multiple phenolic –OH groups, enabling them to interact efficiently with different gelatin chains and thereby form a stable crosslinked network.

#### 3.2.3. XRD

**Figure 3:**
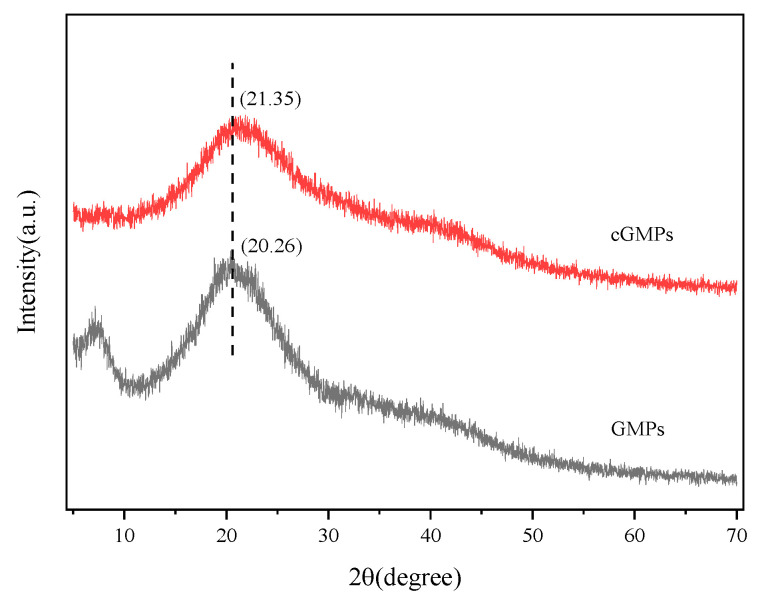
XRD patterns of GMPs and cGMPs.

Figure 3 illustrates the XRD patterns of GMPs and cGMPs, both exhibiting a broad peak at 2θ = 20°. After crosslinking with TP, the characteristic peak of GMPs at 2θ = 7° almost disappeared. This peak position corresponds to the diameter of the triple-helical structure, and its intensity reflects the relative content of this structure [28]. This phenomenon indicates that the crosslinking effect of TP markedly disrupted the triple-helical structure of gelatin. This may be because the multi-point hydrogen bonding between TP and gelatin hindered triple helix reconstruction. In particular, the –OH groups of TP form multiple hydrogen bonds with the peptide bond (–CONH-) of gelatin, occupying the interaction sites necessary for helix formation, thereby preventing the regular folding of peptide chains. This finding corroborates the ATR-FTIR spectroscopy results. The characteristic peak of gelatin at 2θ = 20° is related to the distance between amino acid residues on the helix [29]. After crosslinking, the intensity of this diffraction peak in cGMPs further decreased, likely resulting from the crosslinks formed between gelatin and TP, leading to an additional loss of the triple-helical structure.

#### 3.2.4. DSC

**Figure 4:**
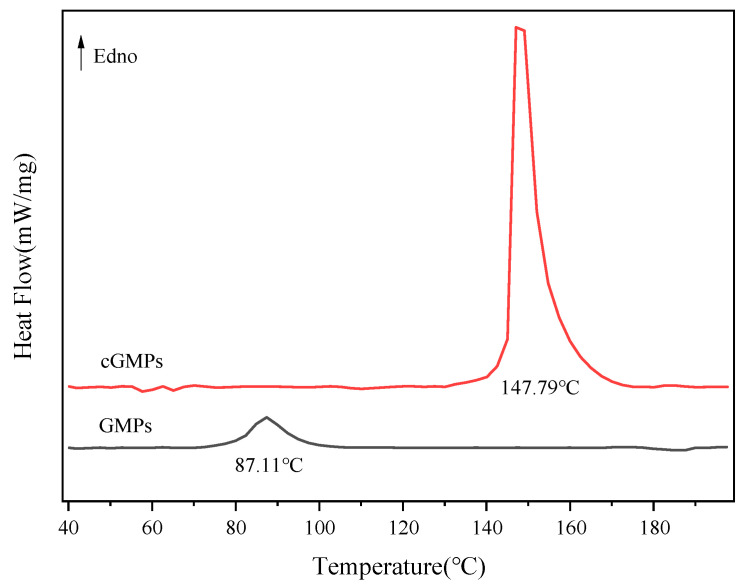
DSC thermograms of GMPs and cGMPs.

The effect of TP crosslinking on microparticles’ thermal stability was investigated using DSC. Figure 4 and Table 1 present the melting behaviour and corresponding energy changes in the microparticles within the temperature range of 40–200 °C. Compared with GMPs, the melting temperature of cGMPs prepared via TP crosslinking markedly increased to 147.79 °C. This result indicates that TP enhances the hydrogen bonding interactions between gelatin peptide chains, thereby improving structural stability. TP–gelatin crosslinking restricts the mobility of gelatin molecular chains and strengthens intermolecular forces. Disrupting these enhanced interactions requires higher energy input, which is reflected in the increased ΔH value (from 15.74 J/g to 100.20 J/g). These findings corroborate the ATR-FTIR spectroscopy (Figure 2) and XRD (Figure 3) results, collectively confirming that the crosslinking effect of TP can effectively enhance the thermal stability of GMPs.

### 3.3. Stability Analysis of FA-cGMPs in High-Temperature Aqueous Solutions

**Figure 5:**
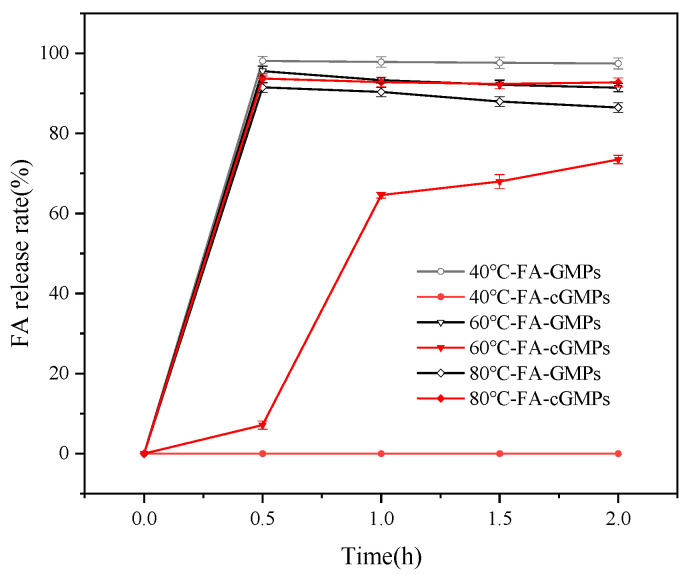
FA release rates in FA-GMPs and FA-cGMPs in 40 °C, 60 °C, and 80 °C aqueous solutions.

**Figure 6:**
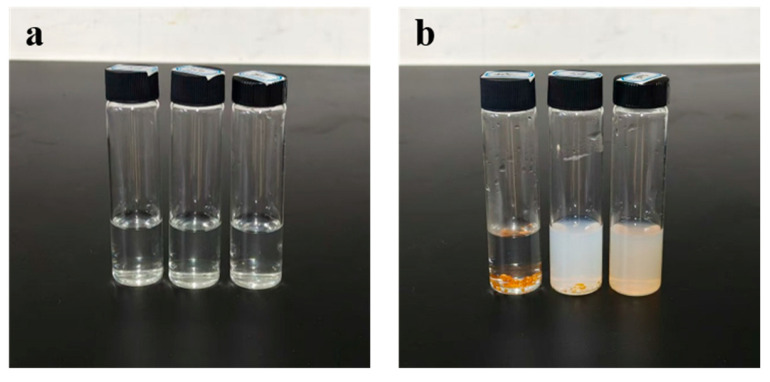
(**a**) shows, from left to right, the remaining state of FA-GMPs after stirring in 40 °C, 60 °C, and 80 °C aqueous solutions for 2 h; (**b**) shows, from left to right, the remaining state of FA-cGMPs after stirring in the same aqueous solutions for 2 h.

Figure 5 and Figure 6 show the FA release rate and storage stability of the samples in the high-temperature aqueous solution. FA was rapidly released as the FA-GMPs matrix dissolved completely within the first 0.5 h of stirring in aqueous solutions at 40 °C, 60 °C, and 80 °C. Following this release, a gradual degradation of the free FA in solution was confirmed by the observed decrease in FA content over time. Specifically, the FA content decreased from 98.12% (±1.14%) at 0.5 h to 97.49% (±1.39%) at 2 h (40 °C), and from 95.56% (±1.19%) at 0.5 h to 91.43% (±1.02%) at 2 h (60 °C). A more pronounced degradation was observed at 80 °C, with the content dropping from 91.51% (±1.22%) at 0.5 h to 86.48% (±1.21%) at 2 h. These consistent downward trends across all temperatures unequivocally demonstrate the degradation of FA under these conditions. For FA-cGMPs, no FA release was observed after 2 h of stirring in the 40 °C solution, with the microparticle structure remaining intact and only slight swelling occurring. In the 60 °C solution, the FA release rate was only 7.11% (±1.01%) at 0.5 h; microparticle rupture occurred at 1 h, resulting in rapid FA release with the release rate increasing to 64.57% (±0.72%) and reaching 73.48% (±1.05%) at 2 h. In the 80 °C solution, complete FA release occurred within the first 0.5 h. Although substantial FA release from FA-cGMPs was observed after 0.5 h of stirring at temperatures above 60 °C, the soluble components still provided certain protection to FA, resulting in higher FA retention than FA-GMPs within 2 h and relatively stable FA content in the aqueous solution. Therefore, FA-cGMPs are suitable in aqueous processing environments below 60 °C.

### 3.4. In Vitro Digestion of FA-cGMPs

**Figure 7:**
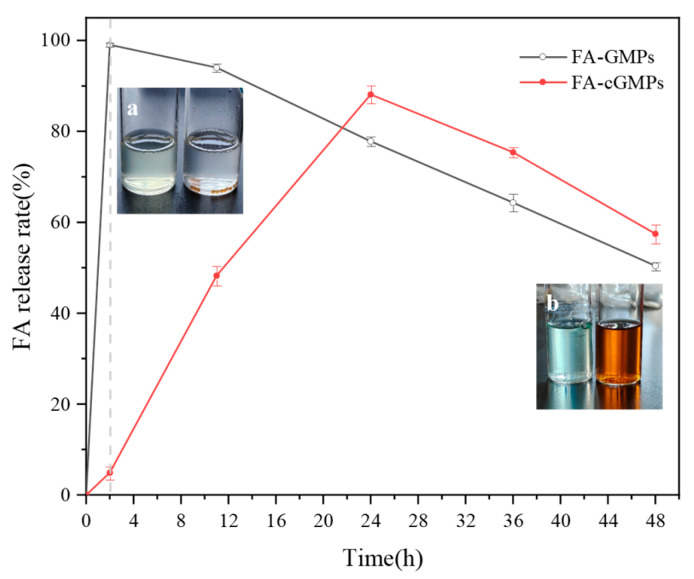
FA release rates in FA-GMPs and FA-cGMPs: (**a**) shows the remaining states of FA-GMPs and FA-cGMPs from left to right after 2-h gastric stage; (**b**) shows the remaining states of FA-GMPs and FA-cGMPs from left to right after 48-h gastrointestinal stage.

The in vitro digestion experiment results (Figure 7) indicated that FA in FA-GMPs rapidly swelled and dissolved in gastric fluid and was nearly completely released (release rate of approximately 99.05% ± 0.46%), which may have resulted from the breakdown of gelatin by pepsin in SGF. Subsequently, the released FA was slowly degraded [30] in the intestinal fluid. In contrast, FA-cGMPs demonstrated excellent sustained-release performance: their release rate in gastric fluid was extremely low (only 4.91% ± 1.41%), mainly due to their dense crosslinked network structure effectively blocking gastric acid erosion. Subsequently, in intestinal fluid, FA-cGMPs achieved highly efficient sustained release (with a release rate of 88.13% ± 1.96%), possibly due to trypsin breaking the crosslinked structure on the microparticle surface, thereby promoting FA release. Overall, FA-cGMPs, as FA delivery vectors, can achieve good sustained-release effects and effectively increase FA bioavailability.

As shown in Figure 7a,b, during the simulated gastrointestinal digestion process, FA-GMPs were completely enzymatically hydrolysed after 2 h of gastric digestion, with the digest appearing light yellow (characteristic colour of FA). After 48 h of complete gastrointestinal simulation, the digest colour changed slightly, presumably attributable to complete protein digestion. In contrast, FA-cGMPs maintained intact spherical structures after 2 h of gastric digestion. Due to the brown colour of the loaded TP, the digest colour transformed from pale white during the gastric phase to brown after intestinal digestion.

## 4. Conclusions

In this study, we prepared cGMPs via TP crosslinking. Compared with GMPs, cGMPs demonstrated significant improvements in anti-swelling capacity, structural stability and thermal stability. SEM analysis revealed that TP crosslinking effectively modified the surface morphology of cGMPs, resulting in a dense and smooth structure. This enhancement resulted from the formation of a sufficient crosslinked network, enabling cGMPs to effectively resist structural collapse and maintain favourable structural integrity during swelling. In contrast, obvious depressions were observed on the GMPs surface. Furthermore, the ATR-FTIR spectroscopy, XRD and DSC results confirmed that TP crosslinking markedly enhanced the structural and thermal stability of cGMPs. Evaluation of high-temperature aqueous stability indicated that FA-cGMPs maintained intact spherical structures for at least 2 h at 40 °C and for 0.5 h at 60 °C. At temperatures exceeding 60 °C, the soluble components of FA-cGMPs still provided certain protection to FA, resulting in higher FA retention than that in FA-GMPs. In vitro digestion demonstrated that FA-cGMPs exhibited excellent sustained-release characteristics, with a release rate of merely 4.91% (±1.41%) in SGF and 88.13% (±1.96%) in SIF, successfully achieving intestinal-targeted release of FA. By comparison, FA-GMPs were almost completely released during simulated gastric digestion. Therefore, FA-cGMPs not only possess outstanding anti-swelling capacity and thermal stability, but also enable targeted intestinal release of FA.

## Figures and Tables

**Table 1:** DSC parameters for GMPs and cGMPs.

Samples	$T_{m}$ (°C)	ΔH (J/g)
GMPs	87.11	15.74
cGMPs	147.79	100.20

## Data Availability

The original contributions presented in the study are included in the article/Supplementary Materials; further inquiries can be directed to the corresponding authors.

## Supplementary Materials

The following supporting information can be downloaded at: https://www.mdpi.com/article/10.3390/polym17212815/s1, Figure S1: Effect of pH on the retention rate of MB-cGMS; Figure S2: Effect of temperature on the retention rate of MB-cGMS; Figure S3: Effect of crosslinking duration on the retention rate of MB-cGMPs; Figure S4: MB standard curve; Figure S5. FA standard curve.

## Abbreviations

The following abbreviations are used in this manuscript:

GMPs	Gelatin microparticles
cGMPs	Crosslinked gelatin microparticles
PVA	Poly(vinyl alcohol)
FA	Folic acid
TP	Tea polyphenols
FA-GMPs	FA-loaded GMPs
FA-cGMPs	FA-loaded cGMPs
MCT	Medium-chain triglycerides
MB	Methylene blue
MB-GMS	MB-loaded GMPs
MB-cGMS	MB-loaded cGMPs
SEM	Scanning electron microscopy
ATR-FTIR	Attenuated total reflection Fourier transform infrared spectroscopy
XRD	X-ray diffraction
DSC	Differential scanning calorimetry
