# Supplementary material for: Preparation of Crosslinked Gelatin Microparticles and Study on Their Loading Capacity for Folic Acid
Source: Polymers (Basel). 2025 Oct 22;17(21):2815. doi: 10.3390/polym17212815 (PMC12608352; doi:10.3390/polym17212815)
Supplement: Supplementary file 1 [file polymers-17-02815-s001.zip › polymers-3889412-supplementary.pdf]

1. Effect of Crosslinking Conditions on MB Retention Rate

1.1 Effect of pH on the Retention Rate of MB-cGMPS（18h，35℃）

The retention rate of MB-cGMPs initially increased and subsequently decreased with rising pH, reaching a maximum value of 96.99% (±1.17%) at pH 4.5.

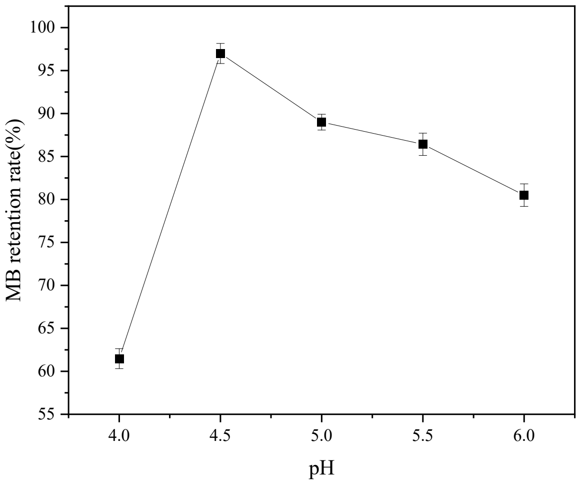

Figure S1. Effect of pH on the retention rate of MB-cGMPs

1.2 Effect of temperature on the Retention Rate of MB-cGMPS（18h，pH4.5）

The retention rate of MB-cGMPs initially increased and then decreased with increasing temperature, reaching a maximum value of 94.17% (±1.00%) at 35 °C, followed by a gradual decline to the minimum value of 47.53% (±1.23%) at 45 °C.

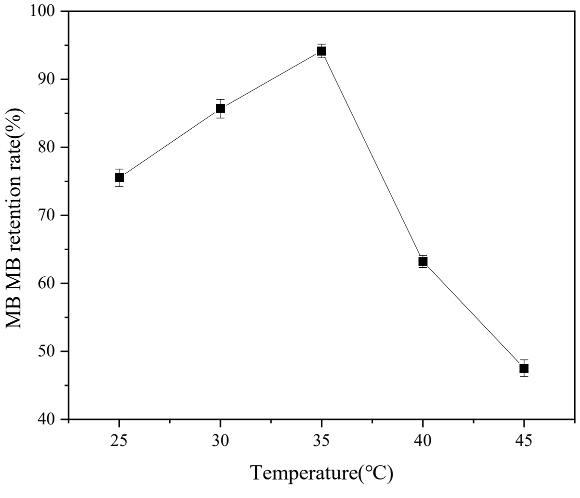

Figure S2. Effect of temperature on the retention rate of MB-cGMPs

1.3 Effect of crosslinking duration on the Retention Rate of MB-cGMPs（pH4.5，35℃）

The retention rate of MB-cGMPs initially increased and subsequently decreased with prolonged crosslinking duration, reaching a maximum value of 94.20% (±0.92%) at t = 18 h, followed by a gradual reduction to the minimum value of 45.57% (±0.97%) at t = 30 h.

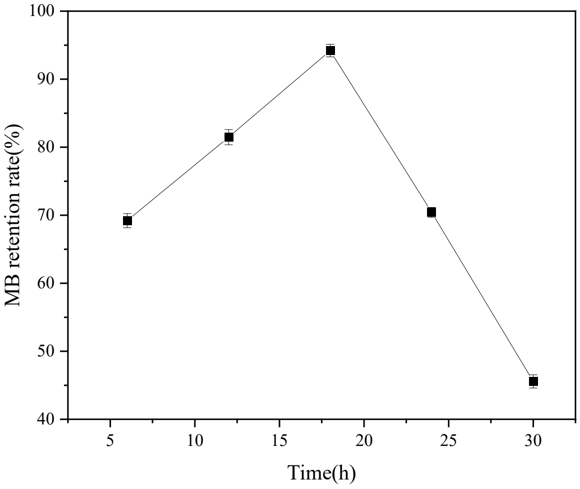

Figure S3. Effect of crosslinking duration on the retention rate of MB-cGMPs

2. Standard curve

2.1 MB standard curve

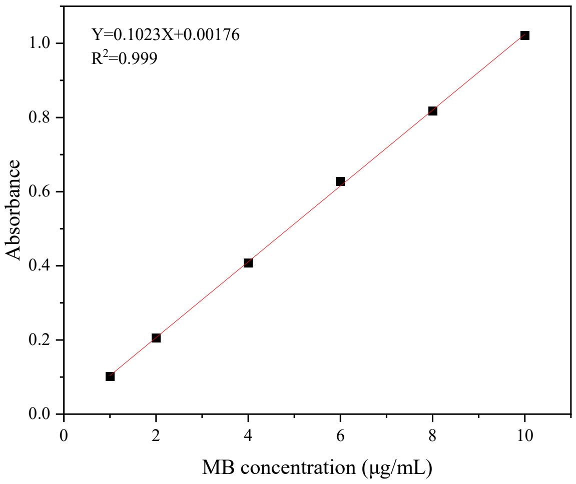

Figure S4. MB standard curve

2.2 FA standard curve

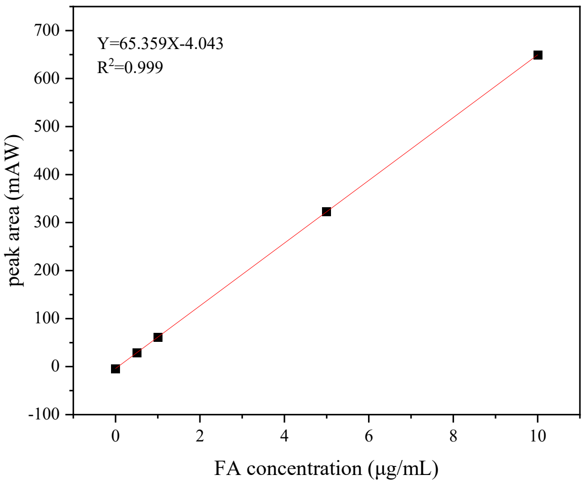

Figure S5. FA standard curve
